# 1162. A data-driven approach to predict plasma leakage using explainable machine learning

**DOI:** 10.1093/ofid/ofac492.999

**Published:** 2022-12-15

**Authors:** Ramtin Zargari Marandi, Preston Leung, Chathurani Sigera, Daniel Dawson Murray, Praveen Weeratunga, Deepika Fernando, Chaturaka Rodrigo, Senaka Rajapakse, Cameron MacPherson

**Affiliations:** Rigshospitalet, Copenhagen University Hospital, Copenhagen Ø, Hovedstaden, Denmark; Rigshospitalet, Copenhagen University Hospital, Copenhagen Ø, Hovedstaden, Denmark; University of Colombo, Colombo, Central Province, Sri Lanka; Rigshospitalet, Copenhagen University Hospital, Copenhagen Ø, Hovedstaden, Denmark; University of Colombo, Colombo, Central Province, Sri Lanka; University of Colombo, Colombo, Central Province, Sri Lanka; University of New South Wales, Sydney, New South Wales, Australia; University of Colombo, Colombo, Central Province, Sri Lanka; Rigshospitalet, Copenhagen University Hospital, Copenhagen Ø, Hovedstaden, Denmark

## Abstract

**Background:**

Dengue could cause complications with an estimated 10,000 deaths per annum. It mostly affects low- and middle-income countries such as Sri Lanka with limited healthcare resources to handle seasonal outbreaks. A third of dengue patients usually have a critical phase characterized by plasma leakage with increased risk of life-threatening complications. A data-driven approach was required to find early predictors of plasma leakage that are usually available in routine care from a resource limited setting, as means of triaging patients for hospital admission.

**Methods:**

We utilized a prospective cohort (The Colombo dengue study in Sri Lanka) that recruits patients meeting the clinical case definition of dengue fever. The cohort includes 4,781 instances of clinical signs, symptoms, and in-hospital laboratory tests from N=877 patients (60.3% patients infected by Dengue) recorded in first four days of fever. By excluding incomplete patient instances, the data was randomly split to a development set (N=378) and a test set (N=144). From the development set, five most informative features were identified using the minimum description length (MDL) algorithm. Logistic regression was used to create a prediction model using the development set. Shapley analysis was used to explain the model on the test set extracting the extent by which each predictor contributed to the predictions.

**Results:**

The MDL algorithm revealed that hemoglobin (HGB), hematocrit (HCT), aspartate aminotransferase (AST), age, and gender to be the most informative predictors of plasma leakage. The logistic regression model predicted plasma leakage with (AUC = 0.76) on the test set (Fig 1). The HGB appeared to contribute the most to the predictions with the higher values associated with higher predicted risk of plasma leakage and vice versa (Fig 2).

**Figure:**
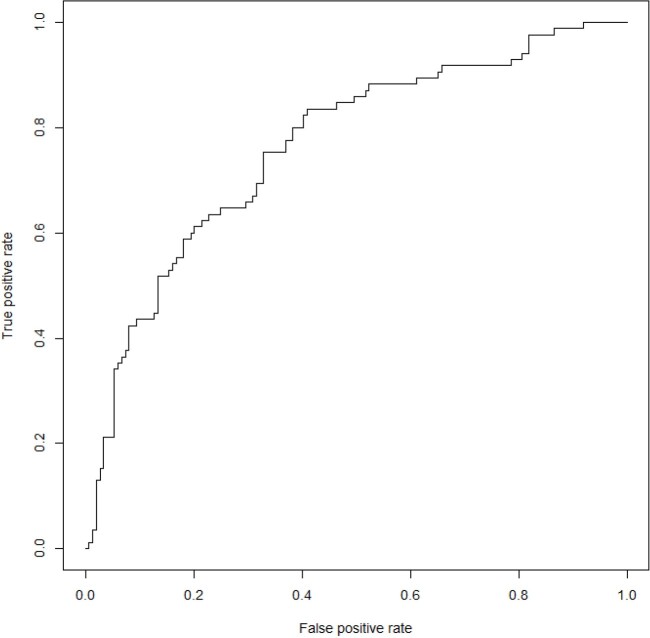

Figure 2 SHAP decision plot for the logistic regression model on the test set. The features are sorted from top to bottom by their mean absolute SHAP values (higher interpreted as more contributing). Feature values are normalised to [0 1] by the min-max normalisation method and colour-coded (grey points are missing values), outliers were squished to the range using Hampel filter. For gender, male is indicated by red and female by blue. The colour of each line is the same as the value of the feature connected to in downwards direction.

**Figure:**
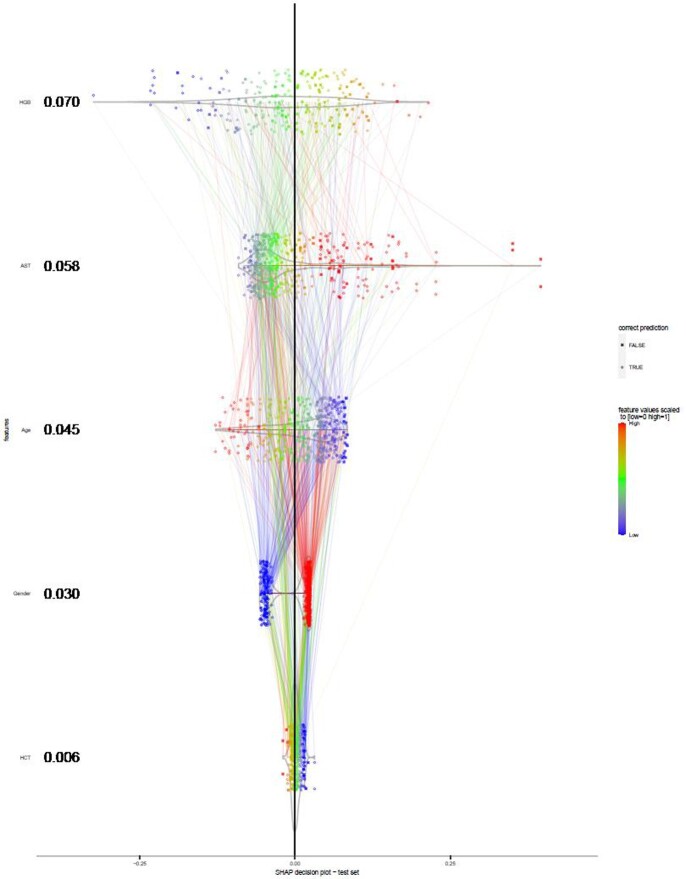

**Conclusion:**

Our results give support to the predictability of plasma leakage in patients suspected of Dengue fever in their first four days of fever onset. The study also underlines the relevance of the machine learning approach to identify the predictors and the practicality of the prediction model as reflected by the prediction performance to triage patients for hospital admission in resource limited settings.

**Disclosures:**

**All Authors**: No reported disclosures.

